# Genome-wide search for the genes accountable for the induced resistance to HIV-1 infection in activated CD4+ T cells: apparent transcriptional signatures, co-expression networks and possible cellular processes

**DOI:** 10.1186/1755-8794-6-15

**Published:** 2013-05-01

**Authors:** Wen-Wen Xu, Miao-Jun Han, Dai Chen, Ling Chen, Yan Guo, Andrew Willden, Di-Qiu Liu, Hua-Tang Zhang

**Affiliations:** 1 Key Laboratory of Animal Models and Human Disease Mechanisms of the Chinese Academy of Sciences & Yunnan Province, Kunming Institute of Zoology, Jiaochang East Road 32, Kunming, Yunnan Province, 650223 China; 2 Chongqing Center for Biomedical Research and Equipment Development, Chongqing Academy of Science and Technology, Chongqing, China; 3 Graduate University of Chinese Academy of Sciences, Beijing, China; 4 Novel Bioinformatics Co., Ltd, Shanghai, China; 5 Yunnan centers for disease control and prevention, Kunming, China; 6 Editorial Department, Kunming Institute of Zoology, Chinese Academy of Sciences, Kunming, China

**Keywords:** HIV-1, Susceptibility, Resistance, CD4 + T cells, CD3/CD28 costimulation

## Abstract

**Background:**

Upon co-stimulation with CD3/CD28 antibodies, activated CD4 + T cells were found to lose their susceptibility to HIV-1 infection, exhibiting an induced resistant phenotype. This rather unexpected phenomenon has been repeatedly confirmed but the underlying cell and molecular mechanisms are still unknown.

**Methods:**

We first replicated the reported system using the specified Dynal beads with PHA/IL-2-stimulated and un-stimulated cells as controls. Genome-wide expression and analysis were then performed by using Agilent whole genome microarrays and established bioinformatics tools.

**Results:**

We showed that following CD3/CD28 co-stimulation, a homogeneous population emerged with uniform expression of activation markers CD25 and CD69 as well as a memory marker CD45RO at high levels. These cells differentially expressed 7,824 genes when compared with the controls on microarrays. Series-Cluster analysis identified 6 distinct expression profiles containing 1,345 genes as the representative signatures in the permissive and resistant cells. Of them, 245 (101 potentially permissive and 144 potentially resistant) were significant in gene ontology categories related to immune response, cell adhesion and metabolism. Co-expression networks analysis identified 137 “key regulatory” genes (84 potentially permissive and 53 potentially resistant), holding hub positions in the gene interactions. By mapping these genes on KEGG pathways, the predominance of actin cytoskeleton functions, proteasomes, and cell cycle arrest in induced resistance emerged. We also revealed an entire set of previously unreported novel genes for further mining and functional validation.

**Conclusions:**

This initial microarray study will stimulate renewed interest in exploring this system and open new avenues for research into HIV-1 susceptibility and its reversal in target cells, serving as a foundation for the development of novel therapeutic and clinical treatments.

## Background

A comprehensive picture of the host factors putatively supporting HIV-1 (human immunodeficiency virus, type 1) replication in cells has emerged from recent siRNA studies [1-4] and meta-analysis [5]. Genome-wide landscapes of host genes and proteins involved in HIV-1 infection and disease progression have also been established in gene array studies [6-9] and novel proteomic approaches [10-12]. In parallel, genome-wide association studies (GWAS) have revealed a set of inheritable genetic variations in large populations related to susceptibility to HIV-1 infection [13-15].

Against these exciting developments, there is still no global view of the host cellular factors that render the target cells resistance to infection, in spite of a few well-studied restriction factors [16-19]. A recent genome-wide screening for novel restriction factors [20] further highlighted this awareness and our interest in establishing a more holistic pictures of the host determinants working against HIV-1 susceptibility.

A crucial starting point for studies of this type is the consideration of sample sources, cell types, and experimental settings. In this regard, CD4 + T cells are the first choice; they are the major cell type amongst all the susceptible targets and reservoirs of HIV-1 infection [21]. Apart from their intrinsic susceptibility, CD4 + T cells’ activation *in vivo* (during the natural courses of HIV-1 infection) and *in vitro* (typically with PHA/IL-2 stimulation) is generally recognized as an absolute prerequisite for the virus to replicate productively [22]. However, rather unexpectedly, Levine et al. (1996) found that activation by co-stimulation with CD28 led to a complete loss of susceptibility to HIV-1 infection in these cells. This phenomenon was subsequently confirmed by several independent groups [23-26]. In spite of the fact that several further studies attempted to explore the potential of using thus-stimulated cells for the treatment of SIV/HIV infection in monkey models and clinical settings, progress in elucidating the underlying cellular and molecular mechanisms seems to have halted since 2002 for unknown reasons.

In the present study, in order to investigate the possible mechanisms of the observed reversal of HIV-1 susceptibility in these activated CD4 + T cells, we first replicated the reported experimental settings [27] and then performed genome-wide expression analysis using Agilent microarrays.

## Methods

### Isolation and stimulation of CD4^+^ T cells

Buffy coats were obtained from healthy donors (Kunming Blood Station) and peripheral blood mononuclear cells (PBMCs) were isolated by Ficool-Hypaque (TBD Sciences) gradient centrifugation. Resting CD4 + T cells were then purified by magnetic negative selection (CD4 + T cell Isolation Kit II, Miltenyi Biotec) and only the aliquots of cells with purity > 95% as determined by flow cytometry were used for further analysis. This study was reviewed and approved by the internal review board of the Kunming Institute of Zoology, Chinese Academy of Sciences (approval ID: RTYX20090910-1, approval date: 2009-09-10). All donors provided written informed consent for participation in this study.

Cells were stimulated as previously reported [27,28]. Briefly, freshly isolated CD4 + T cells were resuspended in RPMI 1640 medium (Gibco) supplemented with 10% heat inactivated fetal bovine serum (Gibco) and 20 mM HEPES (Amresco) and seeded in 6-well plates either at an initial density of 2 × 10^6^ cells/well with human recombinant IL-2 (100 U/ml) and PHA (5 μg/ml) or 0.5 × 10^6^ cells/well with polystyrene beads coated anti-CD3/CD28 antibodies (Dynal beads CD3/CD28 T Cell Expander, Dynal) at a bead to cell ratio of 3:1. Half media were changed every 2 days in CD3/CD28 costimualted CD4 + T cells and every 3 days in PHA/IL-2 stimulated cells. Cells were then cultured at 37°C in a humidified incubator with 5% CO_2_ for 6 days. Cells and the derived RNA samples and data sets were labeled “P”, “R”, and “B”, respectively, according to the PHA/IL-2 stimulated, un-stimulated resting and beads-stimulated settings.

### Flow cytometry

Aliquots of cells to be analyzed were washed with FACS buffer (PBS supplemented with 1% bovine serum albumin (BSA)) and stained for 40 minutes in the dark at 4°C with anti-CD45RO-FITC, anti-CD25-PE, anti-CXCR4, fluorescent dye 5-(and −6)-carboxyfluorescein diacetate succinimidyl ester (CFSE) (Sigma-Aldrich), anti-Ki67(Abcam), anti-CCR5 (Biolegend) and anti-CD69-PE-Cy-5 (BD Biosciences) and appropriate isotype controls. After washing with FACS (Fluorescence Activated Cell Sorter) buffer, cells were fixed with 4% paraformaldehyde and analyzed on a FACS Calibur using Cell Quest and FlowJo 7.6.1.

### Gene expression profiling with Agilent microarrays

Total RNA was extracted from stimulated or un-stimulated CD4 + T cells by TRIzol (Invitrogen) followed by a purification using RNeasy columns (Qiagen) according to the manufacturer’s protocols. The amount and quality of RNA preparations were evaluated on an Agilent 2100 Bioanalyzer with RNA6000 Nano Reagents and Supplies (Agilent).

Quality-checked RNAs were then transcribed with the First-Strand cDNA Synthesis Kit (Agilent) and their expression data obtained using Agilent 4 × 44 K Human Whole-Genome 60-mer oligonucleotide microarrays according to the protocols by the manufacturer. The original microarray data from this study are available at the NCBI GEO database (http://www.ncbi.nlm.nih.gov/geo/) under the accession number GSE34252.

### Analysis of microarray data

The normalized ratio of the gene expression signals was log_2_ transformed and hierarchical clustering was performed with average linkage. The clustered heatmap was visualized using Treeview. The RVM (Random variance model) f-test was applied to filter differentially expressed genes for the different situations. After the significance analysis and FDR (false discovery rate) analysis, we selected the differentially expressed genes according to the p-value and FDR threshold set at *p* < 0.01 and FDR <0.01 [29-31]. And the fold changes of any two groups are more than 2.

Series-Cluster analysis was performed to identify the global trends and model profiles of expression according to signal density under the “P”, “R” and “B” conditions and in the P-R-B sequence. Fisher’s exact test and the multiple comparison test [32,33] were applied to identify the model profiles with probability significantly higher than expected as random.

Gene ontology (GO) analysis [34] was performed to facilitate elucidating the biological implications of unique genes in the significant or representative profiles. GO analysis was used to find the main function of the genes having the same expression trend according to the Gene Ontology. Fisher’s exact test and *χ*^2^ test were applied to identify the significant GO categories and FDR was used to correct the *p*-values.

Gene co-expression networks analysis [35] were performed to track the interactions among the differentially expressed genes, according to their normalized signal intensity in 6 representative profiles. Pearson correlation was applied to each pair of genes and the significantly correlated pairs were used to construct the network [36]. To locate the core regulatory genes in the networks, k-core scoring was introduced to simplify graph topology analysis [37,38]. A k-core of a given gene indicates its hub or nodal status with connection to “k” other genes in a network [37,38]. Accordingly, the genes with largest k-core scores and highest degrees of connection were identified as “key regulatory genes” in a network [39] and those unique to each network were selected as “marker genes” for the cellular status of intrinsic susceptibility or induced resistance to HIV-1 infection.

## Results

### Cellular characterization of stimulated and un-stimulated CD4+ T cells

**Figure 1:**
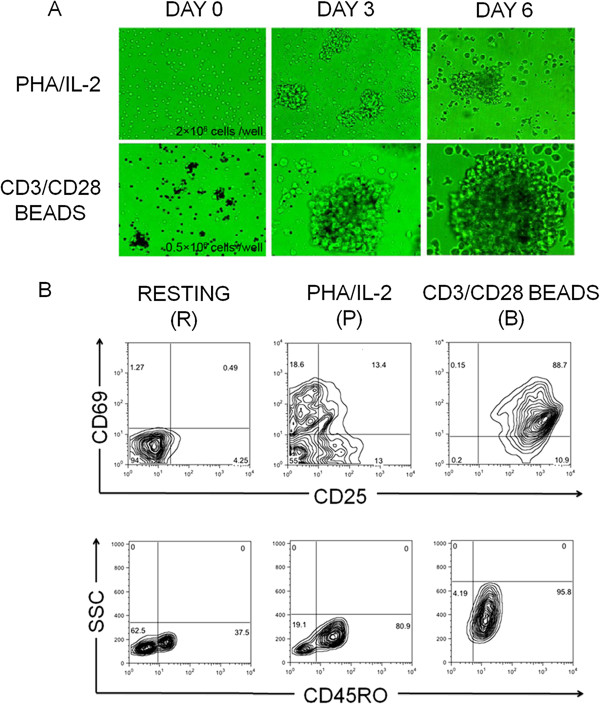
**Morphology and surface phenotypes of stimulated and un-stimulated CD4+ T cells. A**: CD4 + T cells stimulated with PHA/IL-2 (“P”) or CD3/CD28 coated beads (“B”) were observed on day 0 (un-stimulated (“R”)), day 3 and day 6. **B**: Flow cytometric analysis of cell surface phenotypes. “R”, “P” and “B” cells were stained with antibodies to CD25 (FITC), CD69 (PE-Cy5) and CD45RO (FITC). Numbers indicate the percentage of each subset. A majority population of cells emerged in “B” cells which expressed CD25, CD69 and CD45RO. We did the experiments in 19 biological replicates.

Following the previously published details of the initial experiment (Levine et al. 1996) and other independent studies [23-26], we re-established de novo and verified the experimental systems (Figure 1). After stimulation for 6 days with the CD3/CD28 antibodies coated beads (labeled “B” for beads), purified CD4 + T cells became enlarged and highly proliferative, forming a large amount of big cell colonies (Figure 1A). When compared with the un-stimulated (labeled “R” for resting) or PHA/IL-2-stimulated (labeled “P” for PHA) cells at the functional level, the induced resistance to HIV-1 infection in these co-stimulated cells [23-28] was also readily replicated in our earlier experiments (described as a brief report in a home journal [40]). We monitored the cell viability and proliferation first by Trypan blue exclusion test which showed more than 95% viable cells for all the experiments (data not shown) and then by Ki67 and CFSE staining (Additional file 1: Figure S1 and Additional file 2: Figure S2). We then proceeded to analyze the surface phenotypes of the generated cells and found that a homogeneous population of cells emerged following the co-stimulation (Figure 1B). In contrast to the controls, these cells were uniformly and simultaneously stained positive for both the general and early activation markers (CD25 and CD69, respectively) at high levels (Figure 1B). Another important marker for activated and memory T cells, CD45RO, was also uniformly expressed following co-stimulation in these “B” cells but in a much lower percentage than in “R” or “P” cells (Figure 1B).

### Global expression profiles and signatures

**Figure 2:**
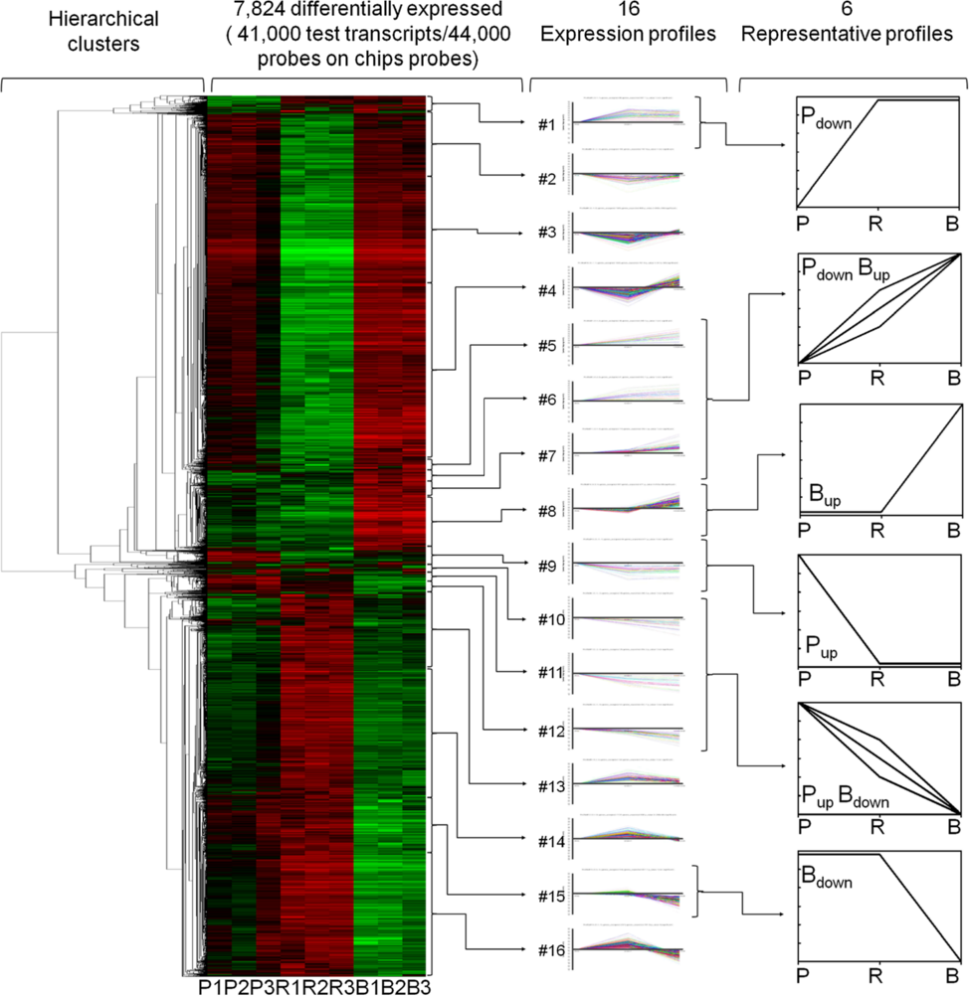
**Overall patterns of 7,824 differentially expressed genes and the 6 representative profiles in the permissive “P” and resistant “B” cells.** Patters were plotted on the heatmap using Treeview. Red represents up-regulated genes while green represents down-regulated genes. Hierarchical clustering is shown on the left. All 16 expression profiles identified by Series-Cluster analysis are shown in the middle and 6 summarized representative profiles are shown on the right.

Using Agilent 4 × 44 K Human Whole-Genome Microarrays, we found that the overall gene expression patterns were clearly and sharply different among the “R”, “P” and “B” cells. As shown in Figure 2 (left) with their clustered heatmaps and hierarchical patterns, in total 7,824 genes represented 8,128 transcripts (out of the 41,000 test probes on the chips) were differentially expressed according to the RVM (Random variance model) algorithm (*p*-value < 0.05, FDR < 0.05) (Additional file 3: Table S1).

In subsequent Series-Cluster analysis, we identified 16 possible profiles (Figure 2 middle) (Additional file 4: Table S2), which represent the overall expression patterns. Of these, 10 (#1, 5, 6, 7, 8, 9, 10, 11, 12 and 15, Figure 2 middle) showed the sharp difference between “B” and “P” cells. More specifically, genes down-regulated in “P” cells are shown in the “P_down_” (#1) profile and those up-regulated in these cells were grouped in the “P_up_” (#9) profile (Figure 2 right). Similarly, genes up- or down-regulated only in “B” are represented in “B_up_” (#8) and “B_down_” (#15) profiles. We merged three profiles (#5, 6 and 7) that contain the genes down-regulated in “P” but up-regulated in “B” into a new “P_down_B_up_” profile, in order to show their special importance in the reduced resistance as they contain the genes which were down regulated in the susceptible “P” cells and at the same up-regulated in the resistant “B” cells. In the same way, we merged profiles #10, 11 and 12 into a new “P_up_B_down_” profile to show the genes that were up-regulated in “P” cells but at the same time down-regulated in the “B” cells (Figure 2 right).

**Figure 3:**
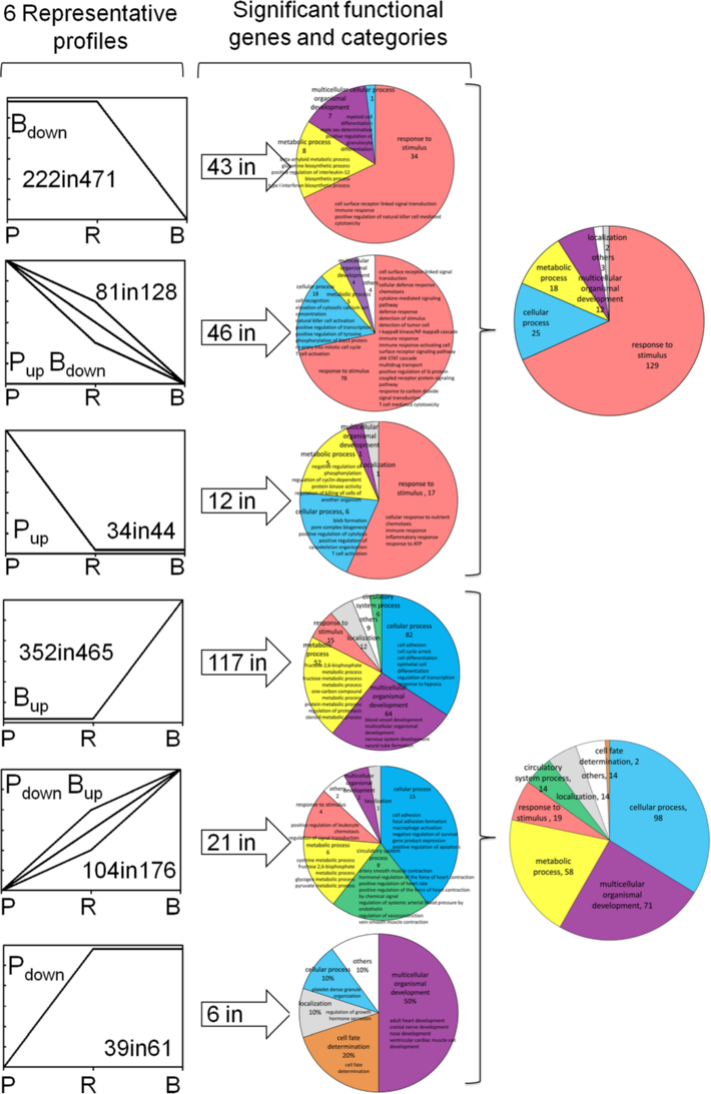
**Gene ontology (GO) analysis and significant functional genes.** Left: Numbers of genes in the 6 representative profiles. Middle: significant functional categories and the number of genes in them. Each color represents one category and the size of each sector in a pie diagram is proportional to the number of genes in its category. Right: Merged functional categories in permissive “P_up_”, “B_down_” and “P_up_B_down_” cells and resistant “P_down_”, “B_up_” and “P_down_B_up_” cells.

These 6 representative profiles contain 1,345 differentially expressed genes in “B” and “P” cells (Figure 3 left) and give the apparent expression signatures either unique to “P” and “B” cells or polarized expressed in “P” and “B” cells at two opposite extremities. The remaining 6,479 genes differentially expressed in the other 6 profiles (#2, 3, 4, 13, 14 and 16, Figure 2 middle) were unique to the “R” cells, up- or down-regulated when compared with “P” and “B” cells as the baseline. Due to the nature of the “R” cells that are seemingly susceptive to the infection but do not support active viral replication, the biological importance of these genes in HIV-1 infection are more complicated to dissect and interpret. We kept aside these 6,479 “R” cell-unique genes for future studies.

### Functional categories and significant ontologies of the differentially expressed genes in “P” and “B” cells

It is immediately noticeable (Figure 3 left) that most of the 1,345 differentially expressed genes in the 6 representative profiles falls in the “B” cell-related expression, with 471 genes in the “B_down_” profile and 465 in “B_up_”, respectively, representing the down-regulated and up-regulated un-permissive genes in the resistant “B” cells (Figure 3 left, Additional file 5: Table S3). In the “B_down_” profile, 222 genes with known functions gave a rather complicated ontology category but, after applying Fisher’s exact test, *χ*^2^ test, and FDR analysis, a clearer picture emerged. Statistically, 43 genes were significantly involved in 4 major functional categories (Figure 3 middle), showing: (a) response to stimulus (immune response, cell surface receptor linked signal transduction and positive regulation of natural killer cell mediated cytotoxicity); (b) metabolic process; (c) multicellular organismal development; and (d) cellular process are predominantly influenced by the co-stimulation.

**Figure 4:**
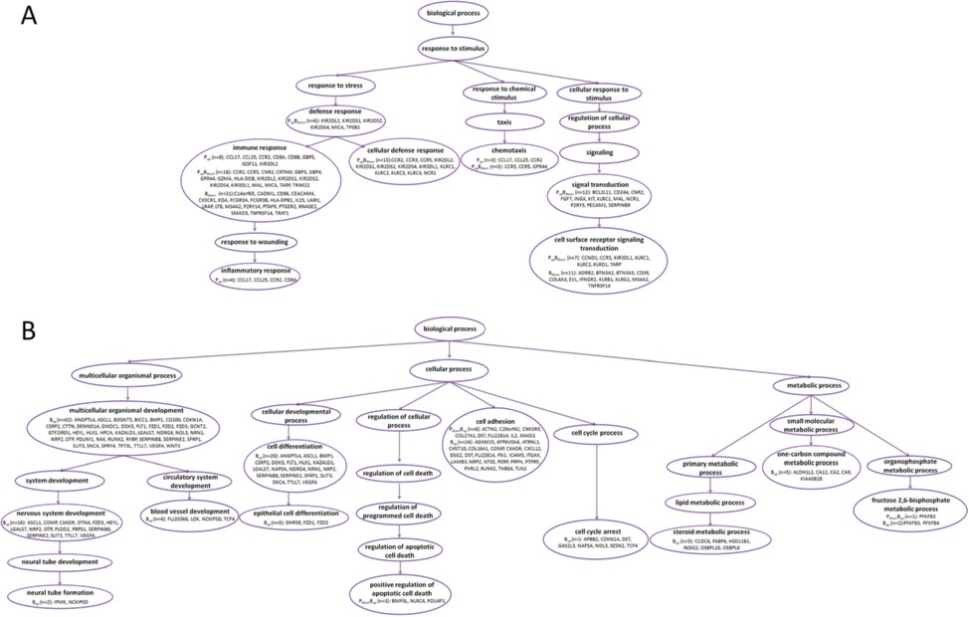
**Hierarchical GO categories of genes involved in induced permissive “P” and resistant “B” cells.** GO categories trees were hierarchically built using the Gene Ontology Enrichment Analysis Software Toolkit (GOEAST): http://omicslab.genetics.ac.cn/GOEAST/). The category tree of response to stimulus related genes in “P_up_”, “B_down_” and “P_up_B_down_” cells is shown in **A**. Category trees of multicellular organismal development, cellular process and metabolic process related genes in “P_down_”, “B_up_” and “P_down_B_up_” cells are shown in **B**.

Other part of exactly the same 4 major categories was found significantly influenced in the “P_up_” and “P_up_B_down_” profiles, with part of one more category (localization) being also up-regulated in the permissive “P” cells or down-regulated in the un-permissive “B” cells (Figure 3 middle). Combined GO analysis of the genes in the “P_up_”, “P_up_B_down_” and B_down_” profiles showed one more category (cell proliferation) was also significantly influenced (Figure 3 right). Altogether, these 6 significantly affected functional categories contain 101 genes (Figure 3 right) representing the whole set of the “permissive genes” that are associated positively with the highly susceptible status or negatively with the induced resistance of the cells. As shown in Figure 4A, these permissive genes are clearly hierarchical and highly enriched in the categories related to defense, immune/inflammatory response and signal transduction under the general heading of response to stimuli [41].

More genes (352) with known functions were identified in the “B_up_” profile with 117 being significantly involved in 7 major GO categories, showing that (a) cellular process (cell adhesion, cell differentiation and regulation of transcription), (b) multicellular organismal development, (c) metabolic process, (d) response to stimulus, (e) localization, (f) circulatory system process, and (g) others are predominantly up-regulated following the co-stimulations. Exactly the same 7 categories were also up-regulated in the “P_down_B_up_” profile, although the numbers of the genes in each category varied (Figure 3 middle right). These 7 categories contain all the 4 significant ones in the “P_down_” profile and one more category (cell fate determination) was also identified. Altogether, the 8 categories contain 144 genes representing the whole set of the “resistant genes” that are associated positively with the induced resistance or negatively with the highly susceptible status of the cells (Figure 3 right). However, they are dispersed in more diverse categories (Figure 4B) than the permissive ones (Figure 4A), indicating that the resistant genes have wider influence in cellular functions and may hold the crucial check points for HIV-1 infection in “multicellular organismal development”, “cellular process” and “metabolic process”. One of the striking examples is that 30 genes involved in the process of cell adhesion were found up-regulated in “B” cells (profiles “B_up_” and “P_down_B_up_”) (Figure 4B) (Additional file 6: Table S4).

### Direct interactions of the genes with known functions with HIV-1 proteins

**Figure 5:**
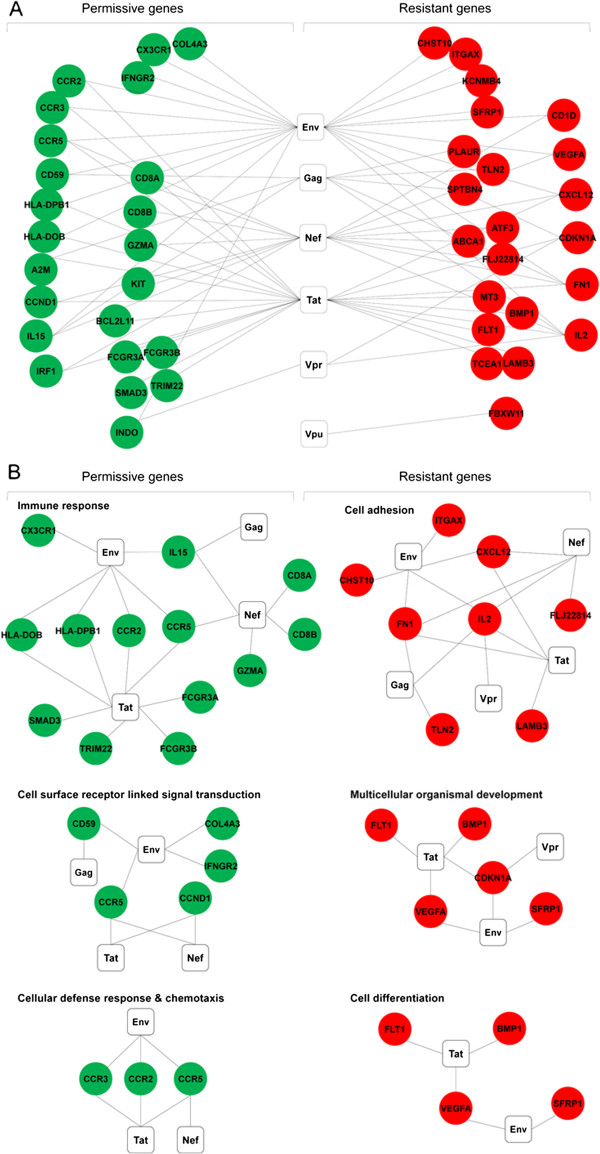
**Interactions of significant functional genes with HIV-1 proteins.** Filled circles represent genes involved in significant functional categories. Red represents genes in profiles “P_down_”, “B_up_” and “P_down_B_up_”, while green represents genes in profiles “P_up_”, “B_down_” and “P_up_B_down_”. Unfilled squares represent HIV-1 proteins. **A**: Overall interactions of significant functional genes with HIV-1 proteins; **B**: Functional categories of known gene interaction with HIV-1.

When subjected to further screening, 22 of the 144 potential “resistant genes” and 23 of the 101 potential “permissive genes” were identified for their direct interactions with HIV-1 proteins (Figure 5A & Additional file 7: Table S5), according to the data in the “HIV-1 Human Protein Interaction Network” (http://www.ncbi.nlm.nih.gov/RefSeq/HIVInteractions/). Their significant functional categories are shown in Figure 5B.

Specifically, 3 (Env, Nef, and Tat) of HIV-1 proteins attracted most of the host (no matter permissive or resistant) factors in this set of data, suggesting that host factors may have the capacity to counterbalance with the permissive genes by interacting with the same early genes in HIV-1 infection. This is coincided with the initial report that the novel anti-HIV effect induced by co-stimulation happens around viral entry and before integration [27]. However, in terms of host gene functions, no overlap was identified between the resistant and permissive genes as the former were mapped on the categories of cell adhesion, multicellular organismal development and cell differentiation, while the latter on immune response, cell surface receptor signal transduction, cellular defense response and chemotaxis. Fewer but prominent genes, e.g., CD59, IL-2, IL-15, INDO and FN1 were found to interact with Gag and Vpr, although we did not find any genes in all our profiles capable of interacting with HIV-1 Vif or Rev.

### Co-expressed genes and their networks in the permissive “P” and un-permissive “B” cells

**Figure 6:**
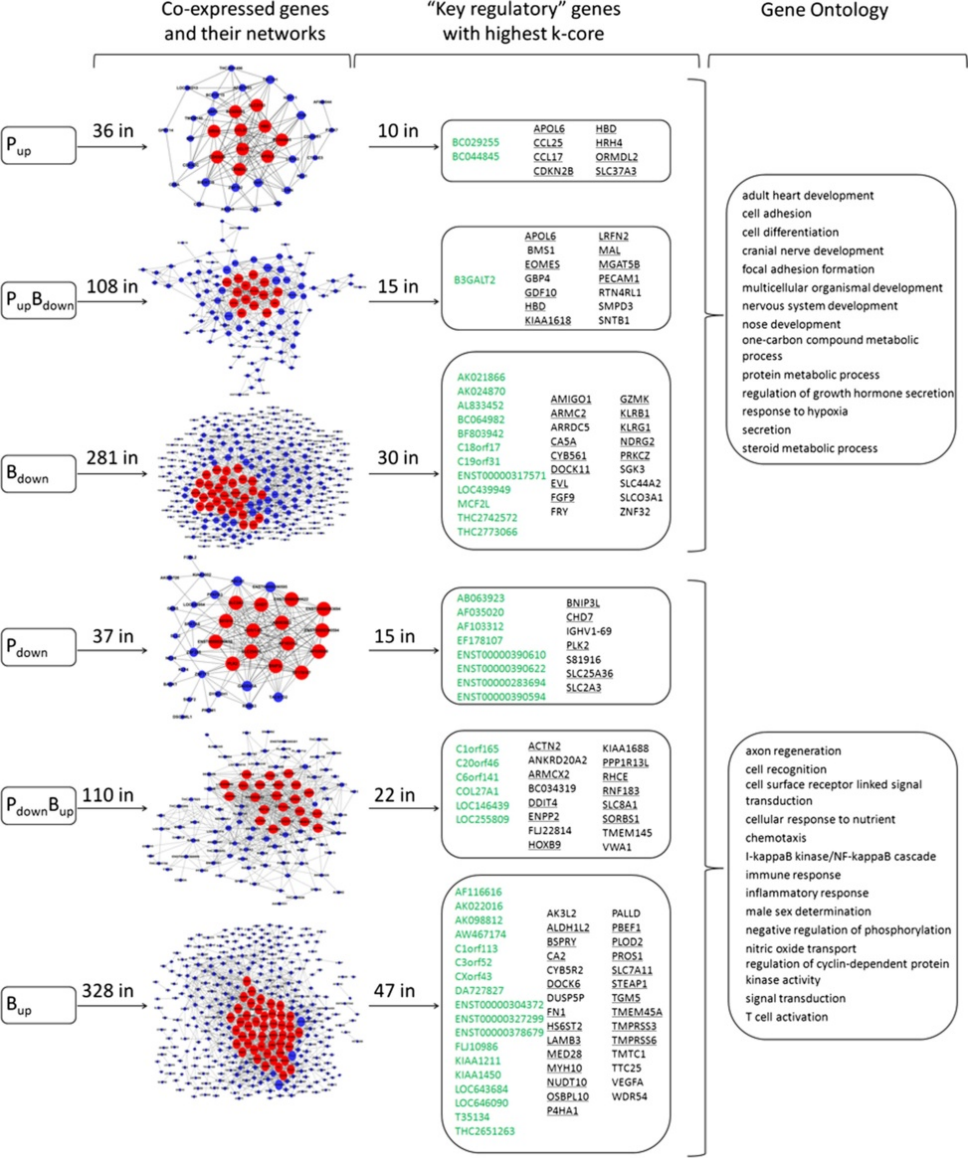
**Co-expressed genes and their networks.** Left: Number of genes in 6 representative profiles (Figure 3 middle left). Red nodes represent “key regulatory” genes while blue nodes represent other regulated genes. Node size represents the power of the interrelation among the nodes, and edges between two nodes represent interactions between genes (i.e. the more edges of a gene, the more genes connecting to it, the more central role it has within the network). Middle right: Name list of “key regulatory” genes with highest k-core. Green represents unknown genes; known functional genes were underlined. Right: significant function of these genes.

Given that HIV-1 tends to interact with “key” host proteins, such as bottlenecks and hubs in gene interaction networks [42,43], we used the genes in the representative profiles and constructed 6 gene co-expression networks (Figure 6 left). We then applied the “k-core” scores (see Methods) to identify those with highest networking degrees as the “key regulatory” genes that may play pivotal roles in gene interactions and regulations. Thus, 53 genes in profiles “P_up_”, “B_down_” and “P_up_B_down_”, which tentatively contain the “permissive genes” were chosen as “key regulatory” genes and, among them, 29 genes with known functions (underlined in Figure 6 middle) are involved in immune response, signal transduction and so on (Figure 6 right). Similarly, 84 genes in profiles “P_down_”, “B_up_” and “P_down_B_up_”, which tentatively contain the “resistant genes”, stood out with the highest k-core scores and, out of them, 36 genes (underlined in Figure 6 middle) are involved in cell adhesion, multicellular organismal development and so on (Figure 6 right).

### KEGG pathways and schematic overview of HIV-host gene interactions in the resistant “B” cells

**Figure 7:**
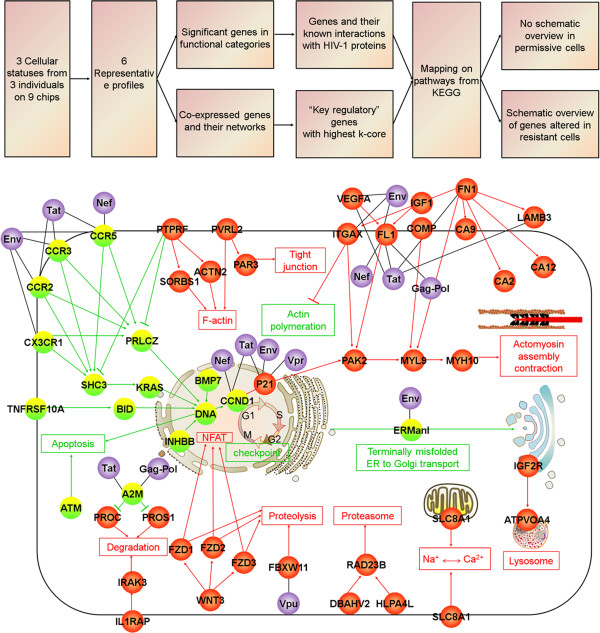
**Schematic overview of the cellular genes altered in “B” cells.** Up: Analysis strategy and results. Down: Schematic network was constructed by 245 genes involved in significant GOs (Figure 3) and 137 genes in co-expression networks (Figure 6) that can also be mapped on KEGG pathways. Processes and genes in profiles “B_up_” and “P_down_ B_up_” or “B_down_” and “P_up_B_down_” are respectively represented by red or green nodes. Purple represents HIV proteins.

**Figure 8:**
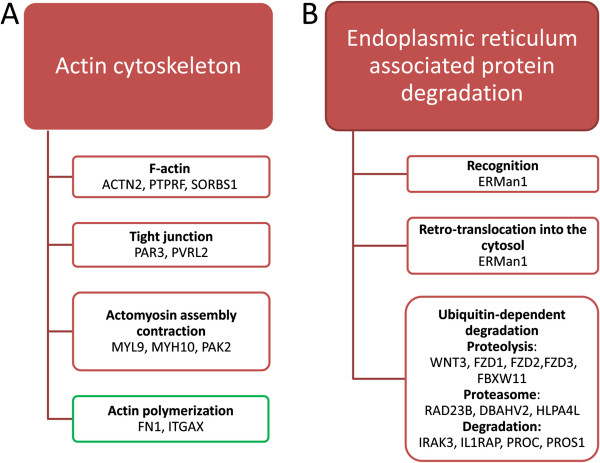
**Actin cytoskeleton and endoplasmic reticulum associated protein degradation related genes in schematic network. A**: Actin cytoskeleton related genes up-regulated in “B” cells. **B**: Endoplasmic reticulum associated protein degradation related genes up-regulated in “B” cells.

We then mapped all the genes with known functions in the significant categories (Figure 3) and all the “key regulatory” genes (Figure 6) on KEGG pathways from the Kyoto Encyclopedia of Genes and Genomes database. A clear picture emerged in the resistant “B” cells showing (Figure 7) that (a) the up-regulated genes in “B_up_” and “P_down_B_up_” profiles were enriched in the cellular processes (Figure 8) including: filamentous actin (F-actin), tight junction, actomyosin assembly contraction, proteasomes, proteolysis, lysosomes, degradation, and Na^+^ Ca^2+^ exchange, and (b) the down-regulated genes in “B_down_” and “P_up_B_down_” profiles were enriched in the process of actin polymeration, apoptosis, cell cycle checkpoint, ER to Golgi transport and terminally misfolded.

### Verification of the differential gene expression by FACS

**Figure 9:**
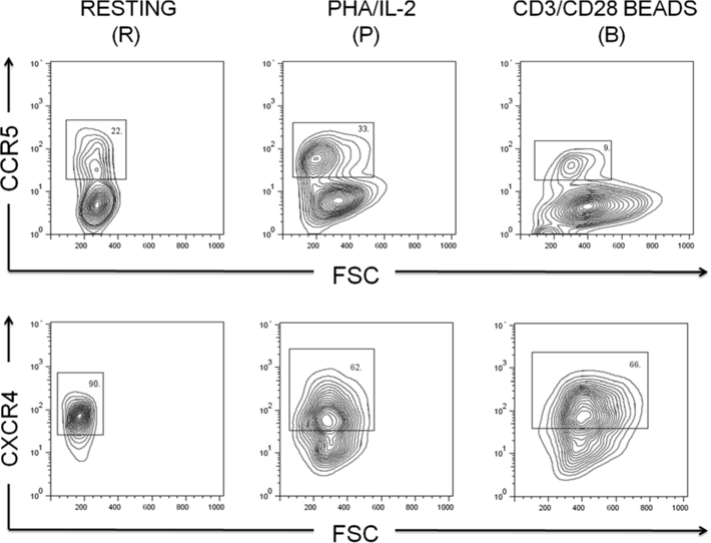
**Verification of CCR5 and CXCR4 expression by flow cytometry.** Cells of “R”, “P” and “B” were stained with antibodies to CCR5 (PE) and CXCR4 (PE) as described in Methods. Numbers indicate the percentage of gated subset that expressed CCR5 or CXCR4. These results have been replicated and confirmed in 10 individuals.

We then performed FACS analysis to examine the expression levels of two HIV-1 co-receptors, CCR5 and CXCR4. As shown in (Figure 9), CCR5 was up-regulated in the permissive “P” cells but down-regulated in the resistant “B” cells, while CXCR4 remained down-regulated in both “P” and “B” cells. These data not only validated our microarray analysis—albeit only a small part of the whole data set—but also were consistent with the initial findings that the induced resistance was only effective to CCR5-dependent strains of the HIV-1 virus [24,44,45]. This also confirmed our previous report [40] showing that, following co-stimulation, the levels of CCR5 expression varied to some degrees in the 12 individuals tested, but the general trend was clearly down-regulated. Conceivably, the down-regulation of the critical co-receptor CCR5 accounts, at least in part, for the induced resistance in the co-stimulated cells.

### Clues for novels genes accounting for HIV-1 susceptibility and resistance

It should be noted that the aforementioned co-expression network analysis was performed using all the genes with and without known functions. Strikingly, among the total 137 “key regulatory” genes, only 9 genes have been previously reported to interact with HIV-1 (Table 1) while the remaining majority (128 genes) as of yet have no confirmation of their potentials to interact with HIV-1 genes or proteins. When going back to the 1,345 differentially expressed genes in the 6 representative profiles (Figure 2 left), around a third of them (513 genes) do not yet have any assigned functions. In view of their prominence in the representative profiles and co-expression networks, these unknown genes constitute a new source for future studies on their roles in biological processes and potential involvement in determining HIV-1 susceptibility and resistance.

**Table 1:** Interactions between HIV-1 proteins and 9 “key regulatory” genes

**Profiles**	**Genes**	**HIV-1 proteins**	**Mode of interactions**
B_up_	FN1	Env	binds
		Gag-Pol	cleaves
		Tat	competes with, modulated by, up-regulates
		Nef	up-regulates
	LAMB3	Tat	up-regulates
	MYH10	Gag-Pol	cleaves
	TGM5	Env	interacts with, modified by
	VEGFA	Env	modulates
		Tat	cooperates with, induces release of
B_down_	PRKCZ	Env	interacts with, inhibits, regulated by, up-regulates
		Gag-Pol	inhibited by, phosphorylated by
		Tat	Activates, regulated by, phosphorylated by
P_down_B_up_	FLJ22814	Nef	up-regulates
P_up_B_down_	MGAT5B	Env	processed by
	SMPD3	Env	activates

## Discussion

In the present study, we re-established an experimental system for cellular and molecular studies on the induced resistance to HIV-1 infection in CD4 + T cells as first reported by Levine et al. [27]. This cellular system includes two methods most commonly used to activate and expand CD4 + T cells *in vitro*. Incubation with PHA/IL-2 induces proliferating CD4 + T cells which are highly susceptible to HIV-1 infection and highly permissive for the subsequent viral replication, whereas co-stimulation with CD3/CD28 reverses the intrinsic susceptibility in these cells and renders them with resistance to HIV-1 infection and un-permissiveness for viral replication. Thus, these two extremely polarized statuses of activated CD4 + T cells, with the un-stimulated resting cells (susceptible to the infection but not permissive for the rapid viral replication) as control in the middle of the “spectrum of permissiveness”, constitute a highly insightful system in the search for host factors responsible for HIV-1 susceptibility and its reversal in CD4 + T cells.

We were attracted more by the biological significance and potentials of the induced resistance in the “B” cells. We first replicated this *in vitro* system and verified its usefulness (Figure 1 and [40]) at the cellular level. Considering the controversy around the source cells, i.e., which subsets (naïve or memory CD4 + T cells) could be induced to generate the resistance [24,26,46], we adopted a strategy using total CD4 + T cell population to start our study without prior cell sorting before co-stimulations. This gave us the chance to address the issue of which subset of CD4 + T cells was generated to be responsible for the induced resistance and led us to the first set of our findings, which shows a homogenous cell population emerged from the co-stimulation. These cells expressed three activation markers (CD25, CD69 and CD45RO) at high levels at the same time. Although the expression of these classical activation markers may not ultimately account for the induced resistance, they served well as indicators for the highly activated population and, more relevant to the following analysis, the homogeneity of the induced cells gave us the confidence for the subsequent whole-genome wide search for the “key regulatory genes” and the “core marker genes” in the status switch to resistance.

Apart from the above novel findings in defining the cell population emerged during the reversal of susceptibility, the present study is the first whole-genome-wide analysis for the genes that account for the induced resistance. Once again, the system allowed us to pin down the potential permissive and resistant genes since susceptible “P” and the resistant “B” cells are polarized at the two extremities of the “permissive spectrum”. As a result, the 1,345 genes in the 6 specific profiles for the “B” and “P” cells could be truly the representatives accounting for the intrinsic susceptibility to HIV-1 infection and its induced reversal in the target cells. This simple dichotomy classification further allowed us to analyze the differentially expressed genes for their gene ontology and co-expression with ease and confidence.

We have not included the popular pathway and transcript factor analysis in this initial genome-wide search but, by focusing on the gene categories and co-expression, managed to identify the major cellular processes involved the reversed susceptibility in the major HIV-1 target cells. Prominently, these include actin cytoskeleton system, protein degradation and cell cycle arrest. First, several lines of evidence highlighted that actin cytoskeleton were regarded as a barrier and the hijack of actin cytoskeleton facilitates entry of HIV into its target cells [47,48]. In our study, 8 genes (ACTN2, PTPRF, SORBS1, PAR3, PVRL2, MYL9, MYH10 and PAK2) were found up-regulated in the resistant “B” cells (“B_up_” profile in Figure 8A). These genes mediate assembly and contraction of actin and actomyosin and formation of tight junctions. Whether they formed a barrier against HIV-1 infection warrant further functional verifications. To the contrary, genes associated with actin polymerization (FN1 and ITGAX, Figure 8A), which have been shown to promote viral binding and entry [48] and is also necessary in chemotaxis and cytokinesis [49] was sharply down regulated in the “B” cells. Given that chemotaxis may actually serve to fuel the infection response by recruiting susceptible, activated CD4 + T cells to the virus, ultimately aiding viral dissemination [49], we therefore hypothesized that co-stimulation could also block the tracks for HIV-1 release and dissemination by reducing actin polymerization and chemotactic response.

Second, genes involved in ubiquitin-dependent degradation by the proteasome (WNT3, FZD1, FZD2, FZD3, FBXW11, RAD23B, DBAHV2, HLPA4L, IRAK3, IL1RAP, PROC and PROS1), which is the third step of endoplasmic reticulum associated protein degradation, were also predominantly in “B” cells (Figure 8B). This is consistent with previously report that the ubiquitin-proteasome was negatively associated with HIV-1 replication by acting to destroy incoming viral complexes at the early steps [50,51]. We also found that ATPVOA4 and IGF2R, which are important for lysosome-mediated degradation of endocytosed proteins [52,53], were up-regulated in “B” cells (Figure 8B). Therefore, co-stimulation could enhance the cellular defense to HIV-1 infection by up-regulating these lysosome- and proteasome-related genes [54].

Third, it is also noticeable that 8 unique genes (Table 2) involved in cell cycle arrest were up-regulated in “B” cells. Apparently, these genes may have helped for the co-stimulated cells to reach the highly proliferative status. However, their role in HIV-1/AIDS infection and disease progression need further detailed studies since modifications to the cell cycles was observed in both long-term non-progressors (LTNPs) and progressive patients [55,56]. Anyhow, this line of evidence reflects that host target cells may have intrinsic mechanisms, which, when activated, would halt viral infection at the specific point of the cell cycle.

**Table 2:** Genes involved in cell cycle arrest

**Profiles**	**Genes of cell cycle arrest**	**GenBank accession**	**Description**
B_up_	APBB2	NM_173075	Homo sapiens amyloid beta (A4) precursor protein-binding, family B, member 2 (Fe65-like) (APBB2), mRNA [NM_173075]
B_up_	CDKN1A	NM_078467	Homo sapiens cyclin-dependent kinase inhibitor 1A (p21, Cip1) (CDKN1A), transcript variant 2, mRNA [NM_078467]
B_up_ & P_down_B_up_	DST	NM_015548	Homo sapiens dystonin (DST), transcript variant 1eA, mRNA [NM_015548]
B_up_	GAS2L3	NM_174942	Homo sapiens growth arrest-specific 2 like 3 (GAS2L3), mRNA [NM_174942]
B_up_	NAPSA	NM_004851	Homo sapiens napsin A aspartic peptidase (NAPSA), mRNA [NM_004851]
B_up_	NOL3	NM_003946	Homo sapiens nucleolar protein 3 (apoptosis repressor with CARD domain) (NOL3), mRNA [NM_003946]
B_up_	SESN2	NM_031459	Homo sapiens sestrin 2 (SESN2), mRNA [NM_031459]
B_up_	TCF4	NM_003199	Homo sapiens transcription factor 4 (TCF4), mRNA [NM_003199]

The other two major cellular programs which were profoundly influenced by the co-stimulation were metabolism and apoptosis (Figure 4B). This seems inconsistent with previous studies showing that (a) numerous metabolism-associated genes were down-regulated in LTNPs [55] and (b) apoptosis is widely accepted as a mechanism for T cell depletion *in vitro* and *in vivo*[3,57,58]. Further 5 genes (CA2, MED28, PLOD2, SLC2A3 and TMTC1), which were up-regulated as probably resistant genes in our “B” cells, were proposed as “host dependent factors (HDF)” in recent small-interfering RNA-knockdown screens [1-4,20]. More surprisingly perhaps, in our set of potentially resistant genes, we did not find any overlap with those reported to be associated with LTNPs [55], viral latency [59,60], or even the “resistance genes” identified by virus-host interaction network analysis using public data sets [61] (data not shown). However, these apparent discrepancies do not necessarily discredit our studies but, to the contrary, may favorably underscore the uniqueness and importance of the highly controlled experimental system we adopted and call for further studies down the line.

Finally, we chose to examine the expression of CCR5 and CXCR4, the 2 major HIV-1 co-receptors, on the cell surface by FACS analysis as the first part of our ongoing efforts to verify our array data and analysis. To our satisfaction, their expression in this set of experiments and our previous screening [40] consistently corroborated our bioinformatics analysis and helped in explaining the fact that the induced resistance by the co-stimulation is a CCR5-dependent phenomenon [24,44,45]. Obviously, large-scale validation and functional studies at both mRNA and protein levels are needed to corroborate the role of the key regulatory genes (e.g., ITGAX, VEGFA, FN1, CCND1, CA12, LAMB3, MYL9, etc.) and their pathways as the major determinants for the induced resistance. We hope the present study serves as a fresh call for a renewed interest in the experimental system as a useful model for the search of novel host resistant factors.

## Conclusions

In summary, we replicated a simple yet powerful cellular system with CD3/CD28 co-stimulation and confirmed its usefulness in studying the induced resistance to HIV-1 in CD4 + T cells. This initial microarray study, although still descriptive and correlative in nature, allowed us a chance to glean valuable new insights into this phenomenon. Based on the overall expression patterns and apparent signatures of the differentially expressed genes, we managed to pin down 245 (101 potentially permissive and 144 potentially resistant) significant functional genes and 137 (84 potentially permissive and 53 potentially resistant) “key regulatory” genes involved in the reversal of target cell susceptibility to HIV-1 infection. Moreover, we showed that this system has the potential as a rich source for the search of novel genes accounting for the intrinsic susceptibility to HIV-1 and its reversal in the major target cells. We hope our findings will stimulate renewed interest in investigating the mechanisms underlying this phenomenon. We surmise that future studies of this kind will help in furthering our understanding of HIV-1 infection, which may ultimately then lead to the development of novel biomarkers and therapeutics.

## Competing interests

The authors declared that they have no competing interests.

## Authors’ contributions

HTZ conceived and designed the study. WWX, MJH & LC performed the experiments. WWX & DC analyzed the data. YG contributed reagents/materials. WWX, DQL & HTZ interpreted the data and wrote the paper. AW revised the manuscript. All authors read and approved the final manuscript.

## Pre-publication history

The pre-publication history for this paper can be accessed here:

http://www.biomedcentral.com/1755-8794/6/15/prepub

## Supplementary Material

Additional file 1: Figure S1 Comparative CFSE staining among “R”, “P” and “B” cells. Cells of “R” were stained with CFSE and cells of “P” and “B” were stained with CFSE on day 3 and day 6. Significant T cell proliferation was observed during the culture period. Click here for file

Additional file 2: Figure S2 Comparative Ki67 staining among “R”, “P” and “B” cells. Cells of “R” were stained with anti-Ki67 and cells of “P” and “B” were stained with anti-Ki67 on day 3 and day 6 and it also showed significant T cell proliferation during the culture period. Red line: isotype control; green line: Ki67. Click here for file

Additional file 3: Table S1 Differentially expressed genes. Click here for file

Additional file 4: Table S2 Profiles of differentially expressed genes. Click here for file

Additional file 5: Table S3 Gene ontologies of the differentially expressed genes in “P” and “B” cells. Click here for file

Additional file 6: Table S4 Hierarchical GO categories of genes involved in induced permissive “P” and resistant “B” cells. Click here for file

Additional file 7: Table S5 Interactions between HIV-1 proteins and significant functional genes. Click here for file
