# Supplementary figures and images for: Genome-wide search for the genes accountable for the induced resistance to HIV-1 infection in activated CD4+ T cells: apparent transcriptional signatures, co-expression networks and possible cellular processes
Source: BMC Med Genomics. 2013 May 1;6:15. doi: 10.1186/1755-8794-6-15 (PMC3655860; doi:10.1186/1755-8794-6-15)

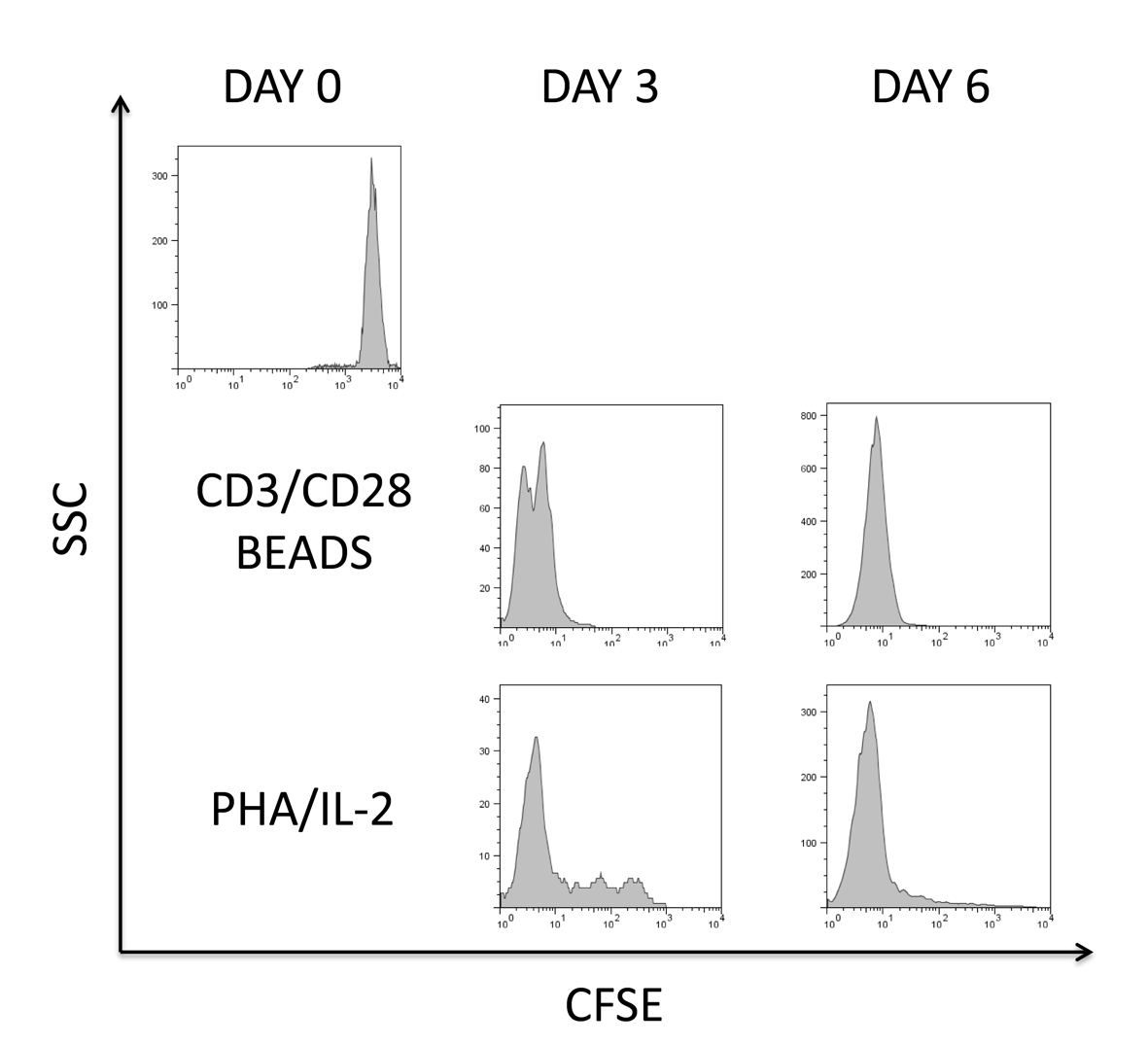

Supplement: Additional file 1: Figure S1 — Comparative CFSE staining among “R”, “P” and “B” cells. Cells of “R” were stained with CFSE and cells of “P” and “B” were stained with CFSE on day 3 and day 6. Significant T cell proliferation was observed during the culture period. [file 1755-8794-6-15-S1.tiff]

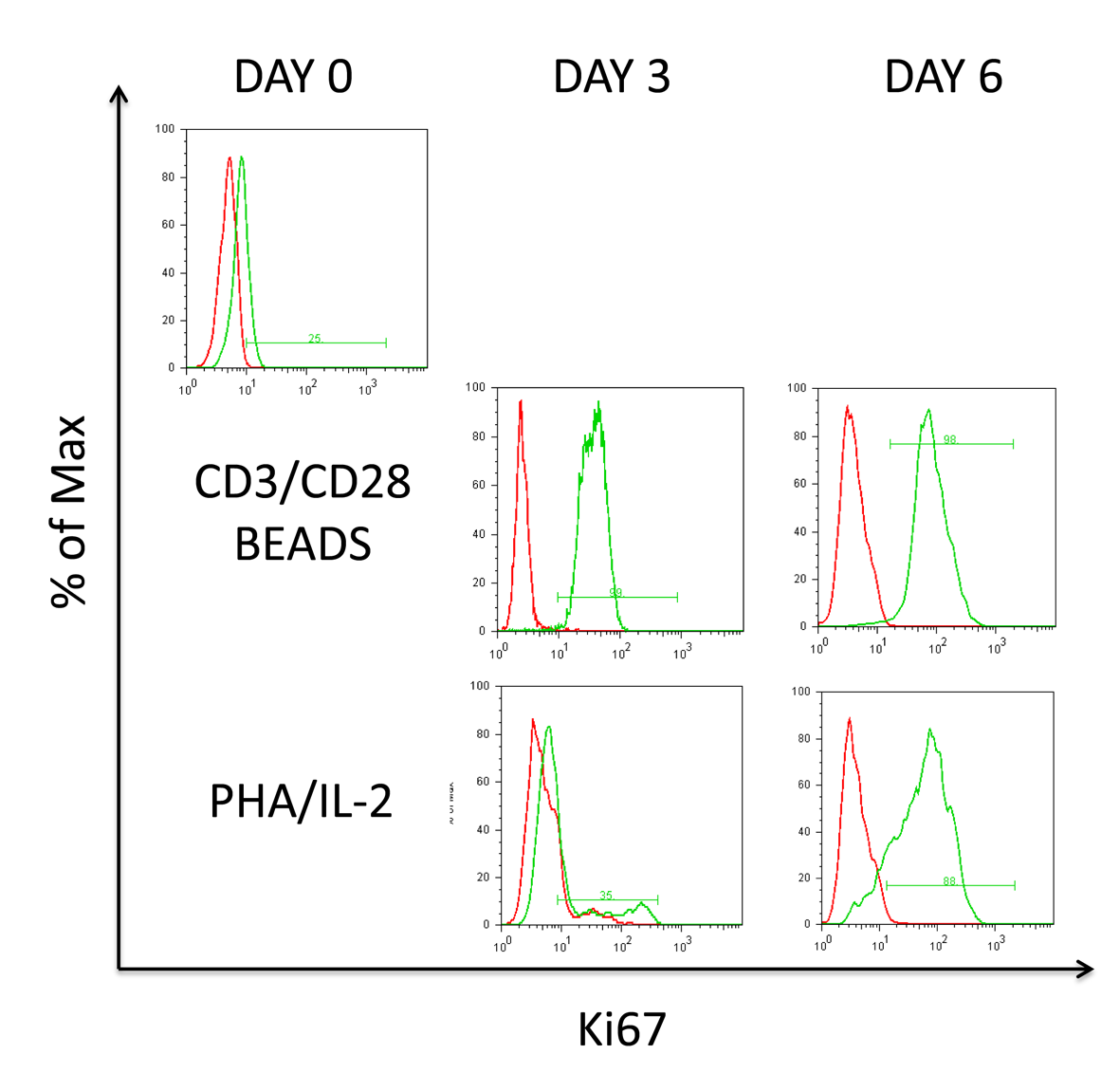

Supplement: Additional file 2: Figure S2 — Comparative Ki67 staining among “R”, “P” and “B” cells. Cells of “R” were stained with anti-Ki67 and cells of “P” and “B” were stained with anti-Ki67 on day 3 and day 6 and it also showed significant T cell proliferation during the culture period. Red line: isotype control; green line: Ki67. [file 1755-8794-6-15-S2.tiff]
